# Supplementary material for: The causal role of circulating inflammatory markers in osteoporosis: a bidirectional Mendelian randomized study
Source: Front Immunol. 2024 Jul 18;15:1412298. doi: 10.3389/fimmu.2024.1412298 (PMC11291241; doi:10.3389/fimmu.2024.1412298)
Supplement: Supplementary file 1 [file DataSheet_1.zip › supplementary material/OSTEOPOROSIS-CIM/GCST90274799.tsv.gz_forest.pdf]

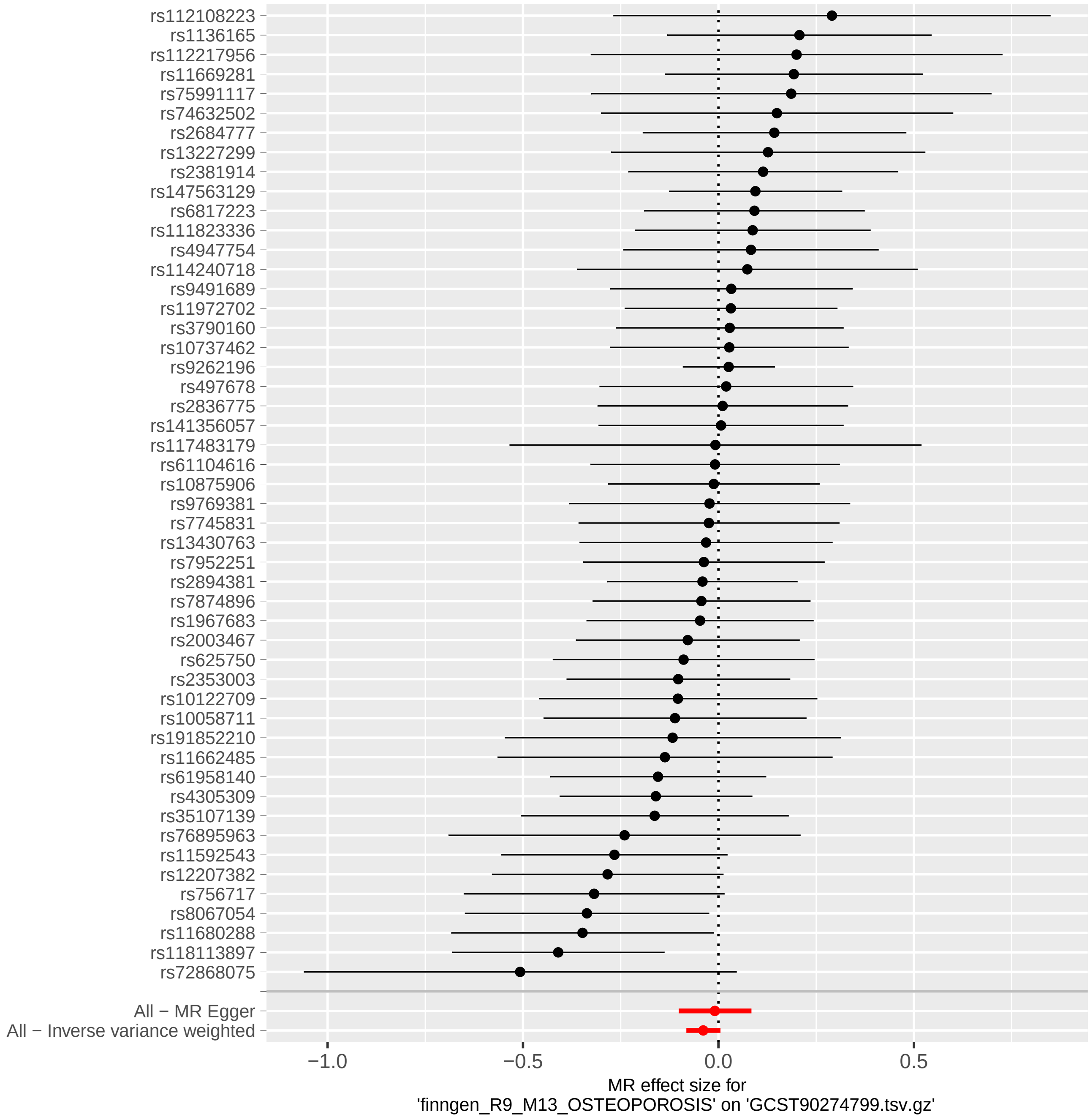
rs112108223
rs1136165
rs112217956
rs11669281
rs75991117
rs74632502
rs2684777
rs13227299
rs2381914
rs147563129
rs6817223
rs111823336
rs4947754
rs114240718
rs9491689
rs11972702
rs3790160
rs10737462
rs9262196
rs497678
rs2836775
rs141356057
rs117483179
rs61104616
rs10875906
rs9769381
rs7745831
rs13430763
rs7952251
rs2894381
rs7874896
rs1967683
rs2003467
rs625750
rs2353003
rs10122709
rs10058711
rs191852210
rs11662485
rs61958140
rs4305309
rs35107139
rs76895963
rs11592543
rs12207382
rs756717
rs8067054
rs11680288
rs118113897
rs72868075
All − MR Egger
All − Inverse variance weighted
−1.0
−0.5
0.0
0.5
MR effect size for
'finngen_R9_M13_OSTEOPOROSIS' on 'GCST90274799.tsv.gz'
